# Current Evidence for Continuous Vital Signs Monitoring by Wearable Wireless Devices in Hospitalized Adults: Systematic Review

**DOI:** 10.2196/18636

**Published:** 2020-06-17

**Authors:** Jobbe P L Leenen, Crista Leerentveld, Joris D van Dijk, Henderik L van Westreenen, Lisette Schoonhoven, Gijsbert A Patijn

**Affiliations:** 1 Department of Surgery Isala Zwolle Netherlands; 2 Department of Intensive Care Isala Zwolle Netherlands; 3 Isala Academy Isala Zwolle Netherlands; 4 Julius Center for Health Sciences and Primary Care University Medical Center Utrecht Utrecht University Utrecht Netherlands; 5 School of Health Sciences, Faculty of Environmental and Life Sciences University of Southampton Southampton United Kingdom

**Keywords:** continuous monitoring, patient monitoring, vital signs, clinical deterioration, early deterioration, wearable wireless device, systematic review, monitoring

## Abstract

**Background:**

Continuous monitoring of vital signs by using wearable wireless devices may allow for timely detection of clinical deterioration in patients in general wards in comparison to detection by standard intermittent vital signs measurements. A large number of studies on many different wearable devices have been reported in recent years, but a systematic review is not yet available to date.

**Objective:**

The aim of this study was to provide a systematic review for health care professionals regarding the current evidence about the validation, feasibility, clinical outcomes, and costs of wearable wireless devices for continuous monitoring of vital signs.

**Methods:**

A systematic and comprehensive search was performed using PubMed/MEDLINE, EMBASE, and Cochrane Central Register of Controlled Trials from January 2009 to September 2019 for studies that evaluated wearable wireless devices for continuous monitoring of vital signs in adults. Outcomes were structured by validation, feasibility, clinical outcomes, and costs. Risk of bias was determined by using the Mixed Methods Appraisal Tool, quality assessment of diagnostic accuracy studies 2nd edition, or quality of health economic studies tool.

**Results:**

In this review, 27 studies evaluating 13 different wearable wireless devices were included. These studies predominantly evaluated the validation or the feasibility outcomes of these devices. Only a few studies reported the clinical outcomes with these devices and they did not report a significantly better clinical outcome than the standard tools used for measuring vital signs. Cost outcomes were not reported in any study. The quality of the included studies was predominantly rated as low or moderate.

**Conclusions:**

Wearable wireless continuous monitoring devices are mostly still in the clinical validation and feasibility testing phases. To date, there are no high quality large well-controlled studies of wearable wireless devices available that show a significant clinical benefit or cost-effectiveness. Such studies are needed to help health care professionals and administrators in their decision making regarding implementation of these devices on a large scale in clinical practice or in-home monitoring.

## Introduction

Continuous monitoring of vital signs of inpatients is a common practice in intensive care, medium care, operation theatre, and recovery ward settings [1]. The goal of continuous vital signs monitoring in these settings is early detection of the clinical deterioration, thereby allowing timely intervention [2,3]. However, once patients are discharged to the general ward, vital signs are only monitored intermittently, often just once or twice daily. Early warning scores have been implemented to guide clinical interpretation, but this value is limited by the intermittent nature of the measurements [4-6]. Serious unexpected adverse events do occur regularly in general wards, especially in high-risk postsurgical or elderly frail patients [7-13]. This incidence of adverse events is expected to increase owing to the aging population, increasing complexity of in-hospital care, increasing pressure to limit health care costs, and increasing shortage of nursing staff. These adverse events may be prevented or mitigated if continuous monitoring of vital signs would be available to facilitate early detection of the deteriorating trends in vital signs, thereby allowing timely interventions [14-16]. An important advantage of continuous monitoring may be the insight in the trends, which can be much more informative and predictive than single deviating values [17-19].

Recent studies have shown that continuous monitoring in combination with automated alerts in case of deterioration improves patient outcomes [17,20-23]. However, for continuous monitoring to be applicable in general wards, it should not lead to decreased mobility of the patient. Therefore, continuous monitoring devices should preferably be portable, wireless, and wearable on an easily accessible body part [18,24]. Such wearable devices also have the potential to be used for continuous monitoring of the vital signs of the patients at home or in rehabilitation centers, thereby possibly leading to reduced length of hospital stay and preventing unplanned readmissions [25].

The technology of wearable wireless sensors for vital signs monitoring is advancing rapidly [26]. Many manufacturers are now developing wearable sensors with different capabilities and different underlying technical specifications and algorithms [27]. The reliability and the accuracy of these devices have often only been demonstrated in healthy volunteers instead of in patients with deviating values [17]. In addition, the scientific evidence regarding the feasibility, effectiveness, and costs of these wearable sensors in clinical practice is still very limited [17,28,29]. Previous reviews on continuous monitoring of vital signs did not focus on wearable wireless devices but rather on conventional nonambulant monitoring [14]. The aim of this study was to systematically review the current evidence on wearable wireless devices for continuous vital signs monitoring by providing a thorough overview of the currently available studies.

## Methods

### Design

We conducted a systematic review of the literature by following the guidelines as outlined in the Cochrane Handbook for Systematic Reviews of Interventions version 6.0 and reported according the Preferred Reporting Items for Systematic Reviews and Meta-Analyses (PRISMA) statement [30,31].

### Eligibility Criteria

Studies were considered eligible for inclusion when they met the following criteria: consisted of participants older than 18 years; evaluated a continuous monitoring device that measured vital signs such as heart rate (HR), respiratory rate (RR), blood pressure (BP), temperature, and blood oxygen saturation (SpO_2_) [16]; used a device that measured ≥2 vital signs; used a device that was wireless and wearable; and published after 2009. This timeframe was chosen to prevent the inclusion of papers on outdated technology. Studies were excluded when the device was not wearable by the patient and the device had no formal approval as a medical device through the Conformité Européenne (CE) mark or Food and Drug Administration (FDA) clearance or both. Furthermore, conference abstracts, review articles, letters, editorials, articles without full texts, and non-English or non-Dutch articles were excluded.

The outcomes of interest were as follows: validation (eg, sensitivity, specificity, limits of agreement [LoA]), feasibility (eg, acceptability, user experiences, system fidelity), clinical outcomes (eg, mortality, length of stay, fail-to-rescue [FTR], intensive care unit [ICU] admission), and costs (eg, cost-minimization, cost-benefit, cost-effectiveness, or cost-utility outcomes) [25,32-35].

For validation studies, the prespecified clinically relevant mean difference and LoA were 10±10 beats per minute for HR, 3±3 breaths per minute for RR, 0.5°C±1.0°C for temperature, 10±20 mmHg for systolic BP, and 3%±5% for SpO_2_. The guidelines for the acceptable mean differences and LoA for continuous monitoring of vital signs are unfortunately lacking.

### Search Strategy

A systematic literature search of PubMed/MEDLINE, EMBASE, and the Cochrane Central Register of Controlled Trials was performed with the last search run on September 6, 2019. In addition, the references of the retrieved studies were manually screened to obtain additional relevant studies. The following keywords were used: vital signs, clinical deterioration, and wireless continuous monitoring. Keywords on outcomes were based on terms about validation, feasibility, clinical outcomes, and cost outcomes. The full search strategy is available in Multimedia Appendix 1. The search string was audited by a clinical librarian and adapted for the individual databases and interfaces as needed. The information about the specifications of the wearable devices was obtained from the manuals and fact sheets of the manufacturers.

### Study Selection

All identified references were checked for duplicates and consolidated in the reference manager software (Mendeley 1.19.5). Titles and abstracts of references were independently screened by 2 researchers against the inclusion and exclusion criteria. Full-text articles of references that matched the inclusion criteria were read independently to determine eligibility. Disagreements were resolved by discussion between the 2 review authors; if no agreement could be reached, the third author was consulted.

### Data Collection Process

A data extraction sheet was developed based on the Cochrane Consumers and Communication review group’s data extraction template and was pilot tested using 5 randomly selected included studies and refined accordingly [31]. One review author extracted the data from the included studies and the second author checked the extracted data. Disagreements were resolved by discussion between the 2 review authors; if no agreement could be reached, the third author was consulted.

### Data Extraction and Synthesis

The following data were extracted for each study: (1) first author, country, year of publishing, aim, design, setting, patient population, sample size, and conflicts of interest; (2) manufacturer and name of the device and type of vital signs measured by the device; and (3) outcomes of the studies divided in previously defined categories: validation, feasibility, clinical, and cost outcomes. The study outcomes were presented for each device.

### Risk of Bias of Individual Studies

For assessing the risk of bias of individual studies, 2 authors independently appraised each study critically. Disagreements in the quality assessment between the authors were solved by discussion until consensus was reached. Owing to the large diversity of the included study designs, 3 different instruments were used. The 2018 version of the Mixed Methods Appraisal Tool (MMAT) was utilized for 5 study designs: qualitative, quantitative randomized controlled, quantitative nonrandomized, quantitative descriptive, and mixed methods [36]. Each category contained 5 criteria with the score range from 0 to 5 of the criteria met. For mixed methods studies, scores were calculated as the lowest score from among the 3 relevant designs (quantitative, qualitative, and mixed methods). A score of 0 to 2 was considered as low, a score of 3 and 4 was considered as moderate, and a score of 5 was considered as high. For diagnostic accuracy study designs, the quality assessment of diagnostic accuracy studies 2nd edition (QUADAS-2) was utilized to assess the risk of bias [37]. QUADAS‐2 consists of 4 domains: patient selection, index test, reference standard, and flow and timing. All domains were assessed for the potential for risk of bias and the first 3 domains, that is, patient selection, index test, and reference standard were assessed for concerns regarding applicability. For economic evaluation studies, the quality of health economic studies (QHES) tool was utilized to assess the quality [38]. The QHES instrument is a validated method for assessing the quality of health economic analyses. It consists of 16 items, each with specific weight values ranging from 1 to 9. Each score is multiplied by the weight to produce a total score, with a maximum score of 100.

## Results

### Study Selection

**Figure 1:**
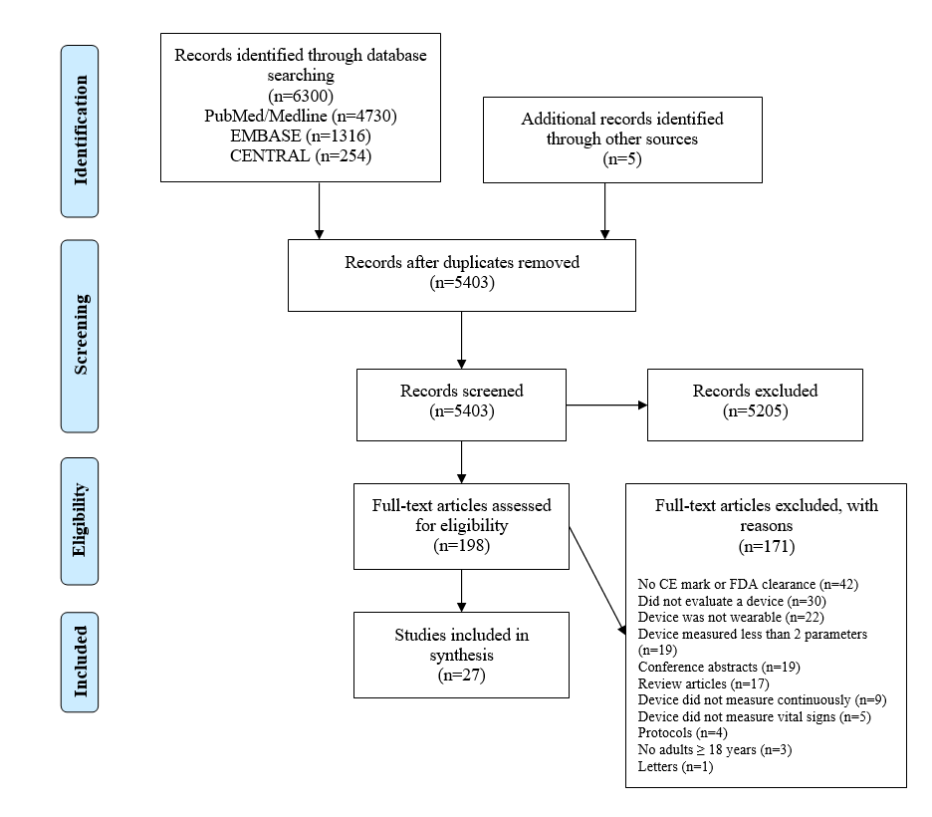
PRISMA flowchart. PRISMA: Preferred Reporting Items for Systematic Reviews and Meta-Analyses; CENTRAL: Cochrane Central Register of Controlled Trials; CE: Conformité Européenne; FDA: Food and Drug Administration.

We identified 5403 potentially relevant studies in our literature search after duplicate removal, of which 5 studies were accessed from the reference list of the potentially relevant studies. Screening of titles and abstracts resulted in 198 studies, which were read full text. Eventually, 27 studies that met the eligibility criteria were included [39-65]. A PRISMA flowchart of the search is presented in Figure 1.

### Study Characteristics

In this study, 13 different devices of 10 manufacturers were studied in 2717 subjects (median 43, range 6-736). Subjects were healthy patients, trauma patients, surgical patients, or neurological/neurosurgical patients (Table 1). The 13 devices were as follows: ViSi Mobile, SensiumVitals, HealthPatch MD, VitalPatch, Wireless Vital Signs Monitor (WVSM) device, MiniMedic, Zephyr BioPatch, Biosensor, IntelliVue Cableless Measurement Solution, Wavelet Wristband, Proteus patch, Alarm Management System, and EQ02 Lifemonitor (Table 2).

Of the 27 included studies, 15 were from the United States and the remaining were from the United Kingdom (N=6), the Netherlands (N=2), Canada (N=1), China (N=1), Australia (N=1), and Austria (N=1). Among these, 13 were validation studies, 6 were cohort studies, 2 were case-control studies, 3 were mixed methods studies, 1 was a qualitative study, and 2 were pilot randomized controlled trials. The reported outcomes were validation (N=15), feasibility (N=15), and clinical outcomes (N=6; Table 3). Seventeen studies declared that they had no conflicts of interest. In 6 studies, one or more authors were employees of the manufacturing company of the studied device. The remaining 4 studies did not declare any possible conflicts of interest (Table 1).

### Devices

#### ViSi Mobile

Five studies (N=1308) have been published about the ViSi Mobile (Sotera Wireless; Table 1) [39,40,51,59,60]. This device is worn on the wrist, upper arm, or chest, and it measures HR, RR, BP, SpO_2_, and skin temperature (Table 2) [66].

*Validation outcomes:* This device was validated in 1 study, which reported an acceptable mean difference but wide LoA between the device and manual nurse measurements for HR, RR, and BP (Table 4) [59]. SpO_2_ had an acceptable mean difference and LoA.

Of the 27 included studies, 15 were from the United States and the remaining were from the United Kingdom (N=6), the Netherlands (N=2), Canada (N=1), China (N=1), Australia (N=1), and Austria (N=1). Among these, 13 were validation studies, 6 were cohort studies, 2 were case-control studies, 3 were mixed methods studies, 1 was a qualitative study, and 2 were pilot randomized controlled trials. The reported outcomes were validation (N=15), feasibility (N=15), and clinical outcomes (N=6; Table 3). Seventeen studies declared that they had no conflicts of interest. In 6 studies, one or more authors were employees of the manufacturing company of the studied device. The remaining 4 studies did not declare any possible conflicts of interest (Table 1).

*Feasibility outcomes:* Patients reported the wristband as big or heavy. Four studies reported the perceptions of the health care professionals [39,51,59,60]. Nurses mentioned that this device had a short battery life and poor connection but it reported better insight into the vital signs [59]. Both nurses and physicians felt confident about their ability to identify patients at risk of deterioration but were concerned about the accuracy of the device [39,59]. Besides, physicians were positive about the potential of continuous monitoring, as this device provided reassurance to patients and supported interdisciplinary communication between nurses and physicians [39]. Another study stated that 67% of the nurses were positive about the deployment of continuous monitoring in the ward [51]. All nurses were positive that the monitor provided valuable patient data that increased patient safety [60]. However, they had certain reservations, including the potential decrease in the bedside nurse-patient contact, increase in inappropriate rapid response team (RRT) calls, and possible discomfort for patients wearing the device [39]. Two studies reported system fidelity. The system generated 2 to 10 alarms per patient in a day [40,60], of which one study [60] reported that 92% of the nurses indicated that the number of alarms were appropriate. One study showed that 70% of the artefacts, defined as the noncollected parameters, were caused by connection failure and 74% lasted less than 5 minutes [59].

**Figure 2:**
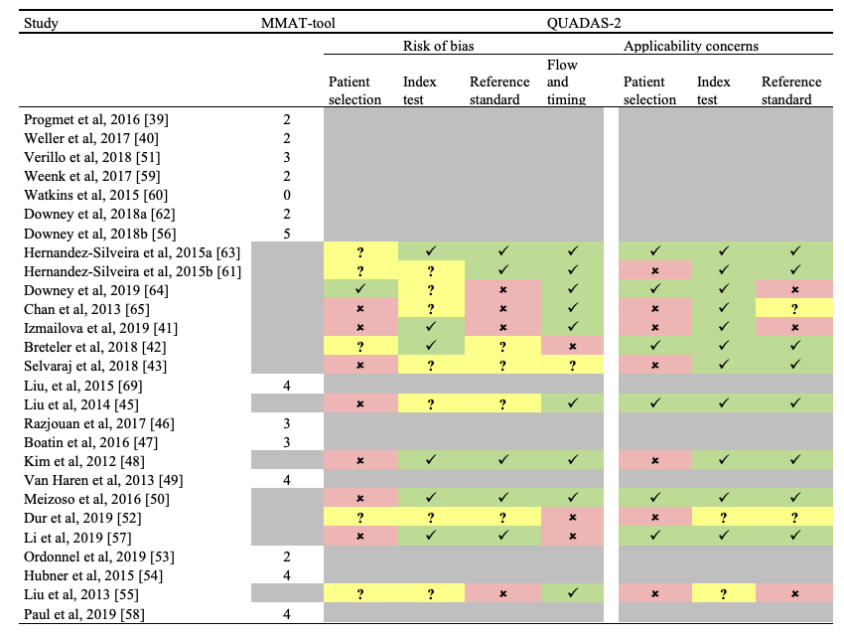
Quality assessment of the included studies. Check marks: low risk of bias; Crosses: high risk of bias; Question marks: unclear risk of bias; Grey cells: Quality assessment tool not used for the study.

*Clinical outcomes:* RRT calls, FTR, unexpected deaths, and ICU transfers were not significantly reduced by continuous monitoring [40,51]. The complication rate was higher in the intermittent monitoring group than in the continuous monitoring group [51]. One study described only 4 alert-initiated interventions in 236 patients [60]. The quality of these studies ranged from low to moderate, as assessed by the MMAT tool, thereby indicating that these studies are subject to bias (Figure 2).

*Cost outcomes:* None of the studies reported this type of outcome.

#### SensiumVitals

Five studies (N=371) have been published about the SensiumVitals (Sensium Healthcare; Table 1) [56,61-64]. This is a patch device attached to the chest for continuous monitoring of the HR, RR, and axillary temperature (Table 2) [67].

*Validation outcomes:* This device was validated in 3 studies. Two studies included surgical patients [63,64] and 1 included healthy volunteers [61]. The results were conflicting. The mean difference between the device and reference standard was acceptable for HR and RR (Table 4). For HR, LoA was acceptable in 2 studies and outside acceptable limits for 1 study. For RR, LoA was wide for all studies. One study [64] reported temperatures outside acceptable ranges. Furthermore, RR was frequently rejected by the algorithm owing to the inaccuracy of the measurement [61,63]. However, the results may be biased owing to the high risk of bias at the reference standards and patient selection (Figure 2). In addition, 2 of the 3 studies [61,63] were authored by the employees of the SensiumVitals manufacturing company and one study was also funded by the manufacturer [61].

*Feasibility outcomes:* Two studies described the feasibility of this device. One qualitative study showed the patient perceptions [56]. Six themes emerged from the interviews: (1) patients emphasized the importance of nursing contact, (2) patients indicated that they hoped to be disturbed less for night-time observations with the new monitoring system, (3) patients reported high comfort, (4) patients experienced a high sense of security, (5) patients expressed that monitoring could be a solution for the busy nursing staff, and (6) patients expressed reservations about the reliability of the technology such as the data security and system failure. The second study reported that patients were comfortable with the patch and that it enhanced the feeling of safety although 16.4% discontinued the intervention owing to the discomfort before the end of the study [62].

*Clinical outcomes:* Only one study reported the clinical outcome. In that study, no statistically significant better clinical outcomes for the patch group were seen, possibly owing to the sample size [62]. Notably, the authors reported that an unacceptable high number of alerts were sent to the nurses before adjusting the alarm thresholds. Since the quality of these studies was rated from low to high by the MMAT tool, possible bias is introduced (Figure 2).

*Cost outcomes:* None of the studies reported this type of outcome.

#### VitalPatch and HealthPatch MD

Five studies (N=133) have been published on the VitalPatch and its previous version HealthPatch MD, which is not available anymore (VitalConnect; Table 1) [41-43,65]. Of them, one mixed methods study compared the HealthPatch with the ViSi Mobile [59]. This patch device is applied to the chest and measures HR, RR, and ST (Table 2) [68].

*Validation outcomes:* This device was validated in 4 studies. For HR, the mean difference was acceptable for all studies and LoA was acceptable for 2 studies (Table 4). The mean difference for RR was acceptable; however, all studies reported LoA outside of the preset acceptable range. One study reported a mean absolute error of less than 3 for HR and 1 for RR [65]. All studies were subject to potential bias at patient selection and the reference standard (Table 4).

*Feasibility outcomes:* The acceptability of this device was reported as high by the majority of the nurses [41]. However, the exact numbers were not reported. Besides, the health care professionals recommended that it was necessary to gain experience with use of the device in clinical practice [41]. Patients reported that the HealthPatch did not restrict them in daily activities. The fidelity of the system was reported in 2 studies, of which one study reported a loss of data of 6% [42]. They compared several thresholds; 63% of the measurements were performed without data loss greater than 2 minutes. In addition, another study reported that more than 50% of all the artefacts lasted for less than 1 minute, and 43% of them lasted for less than 5 minutes [59]. The reasons for these artefacts were wireless signal connection problems or losing skin contact.

None of the studies reported the clinical and cost outcomes.

#### WVSM Device

Two studies (N=305) evaluated the WVSM device (Athena GTX) in trauma patients (Table 1) [45,69]. This device measures the HR, BP, RR, and SpO_2_ continuously and is worn on the chest, upper arm, and fingertips (Table 2) [70].

*Feasibility outcomes:* One study reported the feasibility outcomes [45]. This study was a posthoc analysis of the previous study of Liu et al [69]. They found at least 75% adequate data for BP, HR, and RR for predicting life-saving interventions (LSIs) [45]. However, the results were subject to bias because of a high risk of bias in the following categories: patient selection and flow and timing (Figure 2).

*Clinical outcomes:* One study reported the clinical outcomes and showed that the data of this device were accurate in comparison with that shown in a conventional monitor for the determination of LSIs, without periodic loss of signals or other errors [69]. The authors learned during the study that new medical devices to be used for prehospital studies require integration into the local information technology infrastructure. The quality of this study was rated as high (Figure 2).

None of the studies reported the validation and cost outcomes.

#### MiniMedic

Two studies (N=155) evaluated the MiniMedic (Athena GTX) in trauma patients (Table 1) [49,50]. This device measures the HR, SpO_2_, and ST both at the fingertip and in the forehead (Table 2). In addition, a Murphy factor, an injury acuity algorithm that generates a score, can be calculated for triaged patients in need of LSIs [71].

*Validation outcomes:* One study compared the pulse-wave transit time, a derivate of BP, reported in the device with the BP reported in the conventional monitor and found correlations between them (*R*^2^=0.036, *P*<.001; Table 3) [50]. Temperature measurements were significantly different between the device and the reference standard and between the fingertip and the forehead sensor of the device. For HR, a mean difference of 3 beats per minute was found between the device and the reference standard (*P*<.001). For SpO_2_, the median difference between the conventional monitor and the fingertip sensor was 0% and that between the conventional monitor and the forehead sensor was 7% (*P*<.001). However, this study had a high possibility of bias at patient selection (Figure 2). The second study demonstrated that the MiniMedic was capable of computing a single numeric value, the Murphy factor, to summarize the overall patient status and to identify prehospital trauma patients who need LSIs [49].

None of these studies reported the feasibility, clinical, and cost outcomes.

#### Zephyr BioPatch

Three studies (N=85) have been published about the Zephyr BioPatch (Medtronic Annapolis; Table 1) [46-48]. This is a patch or a patch fixed by a harness on the chest and it measures the HR, RR, and the estimated core temperature (Table 2) [72].

*Validation outcomes:* Two studies reported the validation outcomes (Table 3). One study was conducted in healthy volunteers during graded exercise and in a hot environment and one was conducted in full-term pregnant women [47,48]. For HR, both studies reported acceptable mean differences but nonacceptable LoA. For RR, one study [47] reported acceptable mean differences but nonacceptable LoA for RR but the other study [48] that also reported acceptable mean differences but nonacceptable LoA for RR was subjected to a high risk of bias at patient selection (Figure 2). Therefore, Boatin et al [47] are the only researchers who have reported acceptable mean differences but nonacceptable LoA for RR.

*Feasibility outcomes:* Considering the feasibility outcomes, the participants found the patch comfortable (78%), likeable (81%), and useful (97%). Among nurses, 80% of the nurses found the monitor easy to use and 84% would recommend it to patients [47]. Another study reported a retention rate of 88.6% at the end of the 24-hour monitoring period after exclusion of 2 patients with poor electrocardiogram (ECG) signals [46]. Furthermore, the authors interviewed patients and nurses about any challenges wearing the sensors. Both groups did not report any challenges. The quality of the studies was rated as moderate and high (Figure 2).

None of the included studies reported the clinical and cost outcomes.

#### Biosensor

One study (N=17) reported about the Philips Biosensor, which is a rebrand of the VitalConnect’s HealthPatch (Table 1) [57]. This device is able to measure HR, RR, and ST (Table 2) [73].

*Validation outcomes:* This study only compared the RR of the device with a reference standard. This resulted in acceptable limits of mean difference of 3.5±5.2 breaths per minute and a statistically significant correlation of Spearman’s ρ of 0.86. However, results may be biased due to the high risk of bias regarding patient selection and flow and timing (Figure 2). In addition, 2 authors were employees of Philips and the study was funded by the manufacturer.

This study did not report the feasibility, clinical, and cost outcomes.

#### Wavelet Wristband

One study (N=35) reported about the Wavelet Wristband (Wavelet Health), a watch that monitors HR and RR (Table 1 and Table 2) [52,74].

*Validation outcomes:* For HR, acceptable mean differences and LoA were found (Table 4). For RR, the LoA was outside of the acceptable limits. However, all aspects of risk of bias were either unclear or high and applicability was low (Figure 2). Besides, 4 authors were former or current employees of the manufacturing company.

This study did not report the feasibility, clinical, and cost outcomes.

#### Proteus Patch

We found 1 study (N=13) that reported about the Proteus patch (Proteus Digital Health; Table 1) [53]. This device monitors HR, RR, and ST (Table 2) [75].

*Feasibility outcomes:* In the feasibility study, the patch was able to monitor for over 5 days at home. However, data of 2 patients was insufficient for performing the analysis and were excluded. The quality of the study was rated as low (Figure 2).

This study did not report the validation, clinical, or cost outcomes.

#### IntelliVue Cableless Measurement Solution

We found about the IntelliVue Cableless Measurement Solution (Philips) in 1 study on clinical patients (N=226; Table 1) [54]. This is a device for monitoring the HR, RR, BP, and SpO_2_ (Table 2) [76].

*Feasibility outcomes:* There was an overall good acceptance by patients and health care professionals. No data was lost due to technical difficulties over a median monitoring period of 178 minutes per patient. The quality of the study was rated as high (Figure 2).

This study did not report the validation, clinical, or cost outcomes.

#### Equivital EQ02 Lifemonitor

We found 1 study (N=6) that reported about the Equivital EQ02 Lifemonitor (Hidalgo Ltd) for measuring the HR, RR, ST, and core temperature by using a chest-worn belt monitor (Table 1 and Table 2) [55]. The core temperature was measured using an ingestible pill [77].

*Validation outcomes:* Acceptable results were found for HR and RR (Figure 2). Skin temperature was outside of the acceptable limits for mean difference and LoA, but the core temperature measurement was considered as acceptable. However, these results were subjected to a high risk of bias at patient selection and reference standard (Figure 2).

This study did not report the feasibility, clinical, and cost outcomes.

#### Alarm Management System

We found 1 study (N=250) that reported about the Alarm Management System (Covidien; Table 1) [58]. This device was worn at the fingertip and it measures HR and SpO_2_ (Table 2).

*Feasibility outcomes:* The authors reported that 86.6% of the patients completed the monitoring period in the study. Besides, a mean of 4 alarms per week was reported due to decreased SpO_2_ in about 75% of the alarms.

*Clinical outcomes:* The authors reported respiratory event rates, ICU transfer, and RRT calls. However, this occurred 0 times in the control and 1 time in the intervention group. Eventually, the quality of this study was rated as high (Figure 2).

This study did not report about the validation or cost outcomes.

**Table 1:** Study characteristics.

Author, year	Country	Study design	Setting	Study population	Sample size (N)	Device	Comparison	Conflicts of interest
Prgomet et al, 2016 [39]	Australia	Mixed methods	Single-center hospital	Physicians and nurses of a respiratory and neurosurgery ward	106	ViSi Mobile	None	Not reported
Weller et al, 2017 [40]	USA	Case-control	Single-center hospital	Neurological and neurosurgical patients	736	ViSi Mobile	Manual measurements	None declared
Verillo et al, 2018 [51]	USA	Before-after	Single-center hospital	Orthopedic and trauma patients	422	ViSi Mobile	None	None declared
Weenk et al, 2017 [59]	The Netherlands	Mixed methods	Single-center hospital	Internal and surgical patients	20	ViSi Mobile, HealthPatch	Manual measurements (HR^a^, RR^b^)	None declared
Watkins et al, 2015 [60]	USA	Cohort	2 hospitals	Nurses	24	ViSi Mobile	None	None declared
Downey et al, 2018a [62]	UK	Pilot Randomized control trial	Single-center hospital	General surgical patients	226	SensiumVitals	Manual and intermittent measurements by nurses (HR, RR, temperature)	None declared
Downey et al, 2018b [56]	UK	Qualitative	Single-center hospital	Surgical patients	12	SensiumVitals	None	None declared
Hernandez-Silveira et al, 2015a [63]	UK	Validation study	Single-center hospital	Surgical and comorbid patients	61	SensiumVitals	Philips Intellivue MP30: 3-lead ECG^c^ (HR); Microstream Oridion Capnography (RR)	5 authors were employees of the manufacturing company of the device
Hernandez-Silveira et al, 2015b [61]	UK	Validation study	Laboratory	Healthy subjects	21	SensiumVitals	Rigel 333 patient simulator (HR, RR), Simman (HR), Philips IntelliVue MP30: 2-lead ECG (HR), capnography (RR)	Study was funded by manufacturer, one author was an employee
Downey et al, 2019 [64]	UK	Validation study	Single-center hospital	Major elective surgery patients	51	SensiumVitals	Pulse-oximeter (HR), manually (RR), tympanic thermometer (ST)	None declared
Chan et al, 2013 [65]	USA	Validation study	Laboratory	Healthy subjects	25	HealthPatch	Actiheart, Oridion Capnostream	Authors were employees of the manufacturer of the device
Izmailova et al, 2019 [41]	USA	Validation study	Laboratory	Healthy subjects	6	HealthPatch	Dinamp device (HR), oral thermometer (ST), manual measurement (RR)	None declared
Breteler et al, 2018 [42]	The Netherlands	Validation study	Single-center hospital	Surgical patients	25	HealthPatch	XPREZZON bedside monitor	None declared
Selvaraj et al, 2018 [43]	USA	Validation study	Laboratory	Healthy subjects	57	VitalPatch	Bench testing, Capnostream20, (RR), Actiheart device (HR)	Not reported
Liu et al, 2014 [69]	USA	Validation study	Prehospital	Trauma patients	305	WVSM^d^	LIFEPAK 12 defibrillator/monitor	None declared
Liu et al, 2015 [45]	USA	Cohort	Prehospital	Trauma patients	104	WVSM	None	One author is the CEO^e^ of the manufacturing company
Razjouan et al, 2017 [46]	USA	Cohort	Single-center hospital	Hematology and oncology patients	35	Zephyr BioPatch	None	None declared
Boatin et al, 2016 [47]	USA	Mixed methods	Single-center hospital	Full-term pregnant women and nurses	38	Zephyr BioPatch	Pulse-oximeter (HR), manually (RR)	None declared
Kim et al, 2012 [48]	USA	Validation study	Laboratory	Healthy subjects	12	Zephyr BioPatch	12-lead ECG (HR), Model K4 b2, (RR)	None declared
Van Haren et al, 2013 [49]	USA	Cohort	Prehospital	Patients transported by the prehospital provider	113	MiniMedic	LIFEPAK, Propaq MD monitor	None declared
Meisozo et al, 2016 [50]	USA	Validation study	Single-center hospital	Trauma patients in the intensive care unit	59	MiniMedic	GE Solar 8000M multichannel monitor	Not reported
Dur et al, 2019 [52]	USA	Validation study	Laboratory	Healthy subjects	35	Wavelet Wristband	ECG (HR), spirometry sensor (RR), BIOPAC M36	One author was an employee of Wavelet Health
Li et al, 2019 [57]	USA	Validition study	Single-center hospital	Emergency department	17	Biosensor	Capnography (RR)	Two authors were employees of Philips and study was funded by Philips
Ordonnel et al, 2019 [53]	UK	Cohort	Home	Patients with heart failure	13	Proteus patch	None	None declared
Hubner et al, 2015 [54]	Austria	Cohort	Single-center hospital	Patients at the emergency department and nurses who provided care	226	IntelliVue Cableless Measurement Solution	None	None declared
Liu et al, 2013 [55]	China	Validation study	Laboratory	Healthy subjects	6	Equivital EQ02 Lifemonitor	Polar S810i HR Monitor (HR), Spirometer MLT1000L (RR), MLT422/D TSK probe (Temperature)	Not reported
Paul et al, 2019 [58]	Canada	Pilot randomized control trial	Single-center hospital	Mixed surgical patients	250	Covidien Alarm Management System	None	None declared

^a^HR: heart rate.

^b^RR: respiratory rate.

^c^ECG: electrocardiogram.

^d^WVSM: wireless vital signs monitor.

^e^CEO: chief executive officer.

**Table 2:** Device characteristics.

Device	Manufacturer	Vital signs	Other parameters	Location	BL^a^	CoTy^b^	CR^c^ (meter)	EMR^d^	SoA^e^	D^f^	W^g^	S^h^
ViSi Mobile	Sotera Wireless	HR^i^, BP^j^, RR^k^, SpO_2_^l^, ST^m^	Body posture, fall detection	Upper arm, chest, wrist	14-16 h	Wi-Fi 802.11 radio	180	✓	✓			Clinic
SensiumVitals	Sensium Healthcare	HR, RR, ST	None	Chest, armpit	5 days	Wi-Fi 802.11 b/g	180	✓	✓	✓	✓	Clinic
HealthPatch MD	VitalConnect	HR, RR, ST	HRV^n^, fall detection, step count, body posture, R-R interval, stress level, energy expenditure	Chest	3 days	Bluetooth	max. 10		✓	✓	✓	Clinic, home
VitalPatch	VitalConnect	HR, RR, ST	HRV, steps, body posture, fall detection, activity	Chest	5 days	Bluetooth	max. 10	✓	✓	✓	✓	Clinic, home
Wireless Vital Signs Monitor Device	Athena GTX	HR, BP, RR, SpO_2_	None	Upper arm, chest, fingertip	7+ h	Wi-Fi 802.11 b/g	180	N/A^o^	✓		✓	Clinic, home
MiniMedic	Athena GTX	HR, SpO_2_, ST	PR^p^, PWTT^q^, Murphy Factor	Forehead, fingertip	12 h	Zigbee 802.15.4	100	N/A	✓		✓	Clinic, home
Zephyr BioPatch	Medtronic	HR, RR, estimated CT^r^	Activity, body posture	Chest	12-28 h	Zephyr ECHO gateway, Bluetooth 2.1+, 3G	N/A	N/A	N/A		N/A	Clinic
Biosensor	Philips	HR, RR, ST	Body posture	Chest	4 days	Bluetooth	Max. 10	✓	✓	✓	✓	Clinic, home
IntelliVue Cableless Measurement Solution	Philips	HR, RR, BP, SpO_2_	None	Upper arm, wrist, belly	12-24 h	Short range radio to IntelliVue Guardian Software	<100	✓	✓			Clinic
Wavelet Wristband	Wavelet Health	HR, RR	HRV	Wrist	5 days	Bluetooth	max. 10		N/A		✓	Home
Proteus patch	Proteus Digital Health	HR, RR, ST	None	Upper left chest	7 days	Bluetooth	max. 10	N/A		N/A	✓	Home
EQ02 Lifemonitor	Hidalgo Ltd	HR, RR, ST	ECG^s^, accelerometer, body posture, fall detection	Chest with belt	12-48 h	Bluetooth 2.1, 3G, 4G, GPRS^t^, CDMA^u^	100	N/A	✓		✓	Clinic, home
Alarm Management System	Covidien	HR, SpO_2_	None	Fingertip	N/A	N/A	N/A	N/A	N/A	N/A	N/A	Clinic

^a^BL: battery life.

^b^CoTy: connection type.

^c^CR: connection range.

^d^EMR: electronic medical record.

^e^SoA: system of alerts.

^f^D: disposable.

^g^W: waterproof.

^h^S: setting.

^i^HR: heart rate.

^j^BP: blood pressure.

^k^RR: respiratory rate.

^l^SpO_2_: blood oxygen saturation.

^m^ST: skin temperature.

^n^HRV: heart rate variability.

^o^N/A: not applicable.

^p^PR: pulse rate.

^q^PWTT: pulse wave transit time.

^r^CT: core temperature.

^s^ECG: electrocardiogram.

^t^GPRS: general packet radio service.

^u^CDMA: code-division multiple access.

**Table 3:** Reported outcomes of included studies.

Author, year	Validation outcomes	Feasibility outcomes	Clinical outcomes	Cost outcomes
Prgomet et al, 2016 [39]	—^a^	Knowledge, confidence, perceptions and feedback about continuous monitoring device, interdisciplinary communication regarding deterioration	—	—
Weller et al, 2017 [40]	—	Alarm rate	RRT^b^ calls, ICU^c^ transfers, unexpected deaths	—
Verillo et al, 2018 [51]	—	Staff satisfaction	Complication rate, RRT calls, ICU transfers, FTR^d^ events	—
Weenk et al, 2017 [59]	Bland-Altman agreement	Artifacts, user experiences	—	—
Watkins et al, 2015 [60]	—	Nursing experiences, number of alarms	Log of interventions based on alarms	—
Downey et al, 2018a [62]	—	Patient acceptability and compliance	Time to AB^e^, mortality, length of stay, admission to level II or II, 30-day readmission	—
Downey et al, 2018b [56]	—	Patient perceptions	—	—
Hernandez-Silveira et al, 2015a [63]	Bland-Altman agreement	—	—	—
Hernandez-Silveira et al, 2015b [61]	Bland-Altman agreement	—	—	—
Downey et al, 2019 [64]	Bland-Altman agreement	Completeness of continuous patch data	—	—
Chan et al, 2013 [65]	Mean absolute error, root-mean-square error	—	—	—
Izmailova et al, 2019 [41]	Data collection rate, comparison with control, data limitations	Data collection rate, acceptability	—	—
Breteler et al, 2018 [42]	Limits of agreement and bias	Data loss	—	—
Selvaraj et al, 2018 [43]	Bland-Altman agreement	—	—	—
Liu et al, 2014 [69]	—	—	Prediction of life-saving interventions	—
Liu et al, 2015 [45]	—	Percentages of valid measurements and nonzero waveform samples	—	—
Razjouan et al, 2017 [46]	—	Any potential adverse events or complaints as a result of the patch	—	—
Boatin et al, 2016 [47]	Bland-Altman agreement	Acceptability, functionality	—	—
Kim et al, 2012 [48]	Bland-Altman agreement	—	—	—
Van Haren et al, 2013 [49]	Sensitivity, specificity, negative predictive value, positive predictive value, and area under the receiving operating characteristic curves	—	Prediction of life-saving interventions	—
Meisozo et al, 2016 [50]	Paired student *t*-test, Fisher exact tests	—	—	—
Dur et al, 2019 [52]	Pearson correlation coefficients along with Bland-Altman plots and Bland-Altman limits of agreement	—	—	—
Li et al, 2019 [57]	Correlation, mean difference	—	—	—
Ordonnel et al, 2019 [53]	—	Wear-time detection	Sleep detection	—
Hubner et al, 2015 [54]	—	Monitoring time, patient and user experiences	—	—
Liu et al, 2013 [55]	Bland-Altman agreement, coefficient of variation, ICC^f^, SEE^g^, Pearson correlation coefficients, ANOVA^h^	—	—	—
Paul et al, 2019 [58]	—	Recruitment rate, acceptance and tolerance, number of alarms per day including type and response, reliability of the system	Respiratory event rate, ICU transfer, RRT calls	—

^a^Not available.

^b^RRT: rapid response time.

^c^ICU: intensive care unit.

^d^FTR: fail-to-rescue.

^e^AB: antibiotic administration.

^f^ICC: intraclass correlation.

^g^SEE: standard error of the estimate.

^h^ANOVA: analysis of variance.

**Table 4:** Bland-Altman agreement of validation studies.

Device, study, subgroup		HR^a^, mean difference (Limits of Agreement)	RR^b^, mean difference (Limits of Agreement)	T^c^, mean difference (Limits of Agreement)	SpO_2_^d^, mean difference (Limits of Agreement)	BP syst^e^, mean difference (Limits of Agreement)	BP diast^f^, mean difference (Limits of Agreement)
VitalPatch, Selvaraj et al, 2018 [43]		0.4 (–8.7/9.5)	–1.8 (–10.1/6.5)	—^g^	—	—	—
HealthPatch, Chan et al, 2013 [65]		—	—	—	—	—	—
HealthPatch, Breteler et al, 2018 [42]		−1.1 (−8.8/6.5)	−2.3 (−15.8/11.2)	—	—	—	—
HealthPatch, Weenk et al, 2017 [59]		–1.52 (–12.55/9.51)	–0.64 (10.32/9.04)	—	—	—	—
ViSi Mobile, Weenk et al, 2017 [59]		–0.2 (–11.06/10.66)	1.19 (–5.53/7.91)	—	0.10 (–3.13/3.33)	0.44 (–23.06/23.94)	–8.00 (–27.46/11.46)
**SensiumVitals, Hernandez-Silveira et al, 2015 [63]**							
	Surgical patients	–0.5 (–3.97/2.97)	0.4 (–6.3/7.1)	—	—	—	—
	Cardiovascular disorders (low voltage/variable QRS morphology)	0.97 (–3.73/5.67)	–1.4 (–10.8/8.0)	—	—	—	—
	Cardiovascular disorders (atrial fibrillation)	–1.0 (–8.0/6.0)	–1.0 (–9.4/7.0)	—	—	—	—
	Metabolic disorders	0.9 (–3.5/5.3)	–0.4 (–11.4/10.6)	—	—	—	—
	Diabetes	–0.02 (–6.98/7.02)	0.1 (–7.7/7.9)	—	—	—	—
SensiumVitals, Hernandez-Silveira et al, 2015 [61]		–0.23 (–0.61/0.15)	–0.43 (–6.10/5.20)	—	—	—	—
SensiumVitals, Downey et al, 2019 [64]		1.85 (–23.92/20.22)	2.93 (–8.19/14.05)	0.82 (–1.13/2.78)	—	—	—
Zephyr BioPatch, Boatin et al, 2016 [47]^h^		1.6 (–11.6/14.8) - 4.2 (–4.4/22.8)	0.7 (–4.7/6.1) - 4.2 (–1.9/10.3)	0.02 (–1.48/1.52) - 0.5 (–1.3/2.3)	—	—	—
Zephyr BioPatch, Kim et al, 2012 [48]		0.5 (–15.3/16.3)	–0.6 (–5.6/4.4)	—	—	—	—
Wavelet Wristband, Dur et al, 2019 [52]		–0.3 (–2.6/1.9)	1.0 (–3.0/4.0)	—	—	—	—
Biosensor, Li et al, 2019 [57]		—	3.5	—	—	—	—
Equivital EQ02, Liu et al, 2013 [55]^i^		1.2 (–5.4/7.8)	0.2 (–2.2/2.6)	0.59 (–0.29/1.47; skin) -0.1 (–0.32/0.12; core)	—	—	—

^a^HR: heart rate.

^b^RR: respiratory rate.

^c^T: temperature.

^d^SpO_2_: oxygen saturation.

^e^BP syst: systolic blood pressure.

^f^BP diast: diastolic blood pressure.

^g^Not available.

^h^This study reported the 25th and 75th percentile.

^i^This study reported the Bland-Altman agreement of two types of temperature: skin and core temperature.

## Discussion

### Summary of Evidence

In this study, we aimed to provide a systematic review of the current evidence on wearable wireless continuous monitoring devices for vital signs monitoring. We included 27 studies, which evaluated 13 different wearable devices. Overall, the studies predominantly evaluated the validation of the recorded data (N=15) or the feasibility (N=15) of these devices. Clinical outcomes were only reported in 6 studies, and studies describing the cost outcomes are still lacking. Although 13 different devices were included in this review, these devices did not share the same indication in terms of monitoring. In general, 2 main target indications could be identified. First, the ViSi Mobile, WVSM Device, MiniMedic, and IntelliVue Cableless Measurement Solution were designed for more extensive prehospital (ambulance) or clinical physiological monitoring. This monitoring level may be comparable to standard ICU monitoring, and therefore, these devices are usually bulkier wearable devices. Second, patch, wristband, and harness devices such as the SensiumVitals, VitalPatch, Philips Biosensor, Zephyr BioPatch, EQ02 Lifemonitor, Alarm Management System, Wavelet Wristband, and the Proteus patch were designed for ambulant wireless clinical monitoring of only a few basic vital signs. These devices are possibly more suitable for patients in the general ward and for monitoring the vital signs at home.

Regarding the validation of the devices, a few considerations should be taken into account. Many of these studies were conducted in healthy volunteers, which may introduce a bias owing to the lack of deviating vital signs values when compared to the vital signs of the actual patients. Further, for technical reasons, vital signs cannot be measured continuously by wearable sensors with equal accuracy. In particular, the RR and temperatures still appear to be difficult to be measured reliably in several included studies. In fact, the optimal reference standard for measuring RR has still not been found, although it is considered to be the most important parameter for predicting clinical deterioration [78-81]. In addition, the optimal method for measuring temperature by using wearable wireless devices has yet to be found. Most devices measure the skin temperature, which is known to be unreliable as equivalent for core temperature [82-84].

Feasibility outcomes were focused on acceptability by health care professionals and patients. In general, both groups were positive about the deployment of the devices. In addition, the operation of the system was evaluated, such as the completeness of the measurements and the number and appropriateness of the alarms. Both outcomes were assessed as feasible.

The impact of these devices on clinical outcomes is still unclear because most included studies were underpowered to demonstrate any significant effect. However, multiple studies described cases wherein a complication was recognized earlier by the device and acted upon in a timely manner.

Regarding costs, no outcomes were reported about the devices in the included studies. Such data may however be essential for preparing future business cases for large-scale implementation, considering the relatively high cost of such monitoring devices and platforms [85].

Previously published reviews on continuous monitoring did not focus on wearable devices, except for one, but this was not a systematic review [32]. We found comparable but also contrasting results in that study [32]. The review of Joshi et al [32] reported the same devices as those reported by us as well as some other devices that we excluded since there were no published studies about those devices or they were published before 2009. In line with our results, they also concluded that the diagnostic accuracy of the devices was suboptimal, especially the alarm rates and the false alarms. In addition, they also indicated that there were no sufficiently powered studies to show beneficial clinical effects or cost-effectiveness.

In a review of nonwearable devices, Cardona-Morrell et al [14] found that early detection of deterioration was enhanced but there were no significant improvements in the clinical outcomes, which is in line with our findings regarding wearable devices. This could be explained by the heterogeneous and underpowered character of the included studies [14]. Downey et al [86] also came to this conclusion and further stated that continuous monitoring seems to be feasible in terms of the frequency of implementation in hospitals; they found that patient and nurse perceptions were positive and that continuous monitoring may be cost-efficient.

### Limitations

This systematic review had several limitations. First, the quality varied across the included studies. Several accuracy studies contained high risk of bias regarding patient selection as well as the applicability. Further, the reference standard was often not free from potential bias. Considering the studies assessed with the MMAT tool, quality was predominantly rated 2 or 3 out of 5; therefore, bias is present. Moreover, assessing the quality of the studies and comparing these studies was difficult owing to the heterogeneity of the included studies. Therefore, performing a meta-analysis was not possible owing to the heterogeneity in the devices and the outcomes. Second, 5 of the included studies had possible conflicts of interest owing to funding by the manufacturer or because employees of the manufacturing companies of the devices played a role in the conduct of the study. This highlights the possible risk of reporting and publication bias within this field of research. Third, there were some limitations about the search. We only focused on devices that measured at least two vital signs. However, this cut-off was based on previous studies about the predictive value for clinical deterioration. These studies found that the more vital signs are monitored, the more accurate the detection is [87,88]. Besides, we only focused on off-the-shelf devices with a clearance by the CE mark or FDA as a medical device for clinical use. We excluded 42 prototype studies that were considered to be less clinically relevant for health care professionals. However, this indicates that there may be many more monitoring devices that will be launched in the health care market in the future. Besides, the review was restricted to English and Dutch publications published from 2009 and after. Only a few studies were excluded based on language and the older studies were considered be less clinically relevant owing to outdated technology. Fourth, we prespecified the clinically relevant mean difference and LoA for vital signs. It may be clinically desirable to redefine acceptable accuracy limits depending on the value of the vital signs measured and the patient population. For example, a difference of 3 breaths per minute is more clinically relevant in a range of 5-8 breaths per minute than with 30-33 breaths per minute. However, reliable evidence or guidelines for continuous monitoring of vital signs are currently lacking.

### Clinical Implications

This review outlines several important clinical implications before health systems may proceed to large-scale implementation of wearable wireless continuous monitoring devices for vital signs monitoring for patients in the hospital and at home. For both settings, vital signs data measurements should be accurate, reliable, and validated in clinical studies. This is especially important for the home setting, wherein a health care professional is not readily available to assess the clinical condition of the patient. For further optimization, the monitoring measurements should preferably be incorporated into an early warning score system supported by a validated decision support algorithm [89]. These analysis algorithms should be further enhanced to prevent too many alarms in order to avoid alarm fatigue [90]. Further, for optimal adoption into clinical workflows, the vital signs measurements should preferably be integrated into the electronic medical record. This will likely improve commitment and compliance from nurses and doctors and will also allow for the summarized monitoring data to be archived in the patient records [32]. When all such factors are optimized, it is anticipated that studies will be able to show a significant effect on clinical outcomes. For monitoring patients at home, the patient data need to be sent to health care professionals through a stable and secure wireless connection. Such a system will need to be embedded in a validated care work flow, thereby providing alarm reviews by care professionals who will assess, make an initial phone call, and then escalate to a home visit by a nurse or direct the patient to the emergency department when needed [91]. Furthermore, for home monitoring, the devices should be small, flexible, and hypoallergenic and not bother patients during their daily activities [18,24]. Battery life, which currently ranges from 3 to 7 days in most devices, may be further extended especially for long-term monitoring of patients with chronic diseases such as heart failure [18,19]. Eventually, when all the conditions are optimized, larger studies may be able to demonstrate that continuous home monitoring safely allows for routine early discharge from the hospital. Further, such a system may potentially provide timely detection of complications, and thereby prevent readmissions, improve overall outcomes, and decrease health care costs [21,92].

### Conclusions

Continuous monitoring devices are mostly still in the validation and feasibility phases. Besides, studies reporting clinical outcomes are still sparse and cost outcome studies are still lacking. Such studies are needed to help health care professionals and administrators in their decision making regarding the implementation of these devices on a large scale in clinical practice or in home monitoring.

## Appendix

Multimedia Appendix 1 Search string of PubMed/MEDLINE database.

## Abbreviations

BP: blood pressure
CE: Conformité Européenne
ECG: electrocardiogram
FDA: Food and Drug Administration
FTR: fail-to-rescue
HR: heart rate
ICU: intensive care unit
LoA: limits of agreement
LSI: life-saving intervention
MMAT: mixed methods appraisal tool
PRISMA: Preferred Reporting Items for Systematic Reviews and Meta-Analyses
QHES: quality of health economic studies
QUADAS-2: quality assessment of diagnostic accuracy studies 2nd edition
RR: respiratory rate
RRT: rapid response team
SpO2: blood oxygen saturation
WVSM: wireless vital signs monitor

## References

[1] No authors listed (1999). Guidelines for intensive care unit admission, discharge, and triage. Task Force of the American College of Critical Care Medicine, Society of Critical Care Medicine. Crit Care Med.

[2] Padilla RM, Mayo A (2018). Clinical deterioration: A concept analysis. J Clin Nurs.

[3] Churpek MM, Yuen TC, Edelson DP (2013). Predicting clinical deterioration in the hospital: The impact of outcome selection. Resuscitation.

[4] Downey C, Tahir W, Randell R, Brown J, Jayne D (2017). Strengths and limitations of early warning scores: A systematic review and narrative synthesis. International Journal of Nursing Studies.

[5] Clifton L, Clifton DA, Pimentel MAF, Watkinson PJ, Tarassenko L (2014). Predictive Monitoring of Mobile Patients by Combining Clinical Observations With Data From Wearable Sensors. IEEE J. Biomed. Health Inform.

[6] Credland N, Dyson J, Johnson MJ (2018). What are the patterns of compliance with Early Warning Track and Trigger Tools: A narrative review. Applied Nursing Research.

[7] Perman SM, Stanton E, Soar J, Berg RA, Donnino MW, Mikkelsen ME, Edelson DP, Churpek MM, Yang L, Merchant RM, Nichol G, Nadkarni VM, Peberdy MA, Chan PS, Mader T, Kern KB, Warren S, Allen E, Eigel B, Hunt EA, Ornato JP, Braithwaite S, Geocadin RG, Mancini ME, Potts J, Truitt TL (2016). Location of In‐Hospital Cardiac Arrest in the United States—Variability in Event Rate and Outcomes. JAHA.

[8] de Vries EN, Ramrattan MA, Smorenburg SM, Gouma DJ, Boermeester MA (2008). The incidence and nature of in-hospital adverse events: a systematic review. Qual Saf Health Care.

[9] Fried LP, Tangen CM, Walston J, Newman AB, Hirsch C, Gottdiener J, Seeman T, Tracy R, Kop WJ, Burke G, McBurnie MA (2001). Frailty in Older Adults: Evidence for a Phenotype. The Journals of Gerontology Series A: Biological Sciences and Medical Sciences.

[10] Maurits EEM, de Veer AJE, Francke AL (2016). Ervaringen van verpleegkundigen, verzorgenden, begeleiders en praktijkondersteuners. NIVEL.

[11] Benjamin E, Virani S (2018). Heart Disease and Stroke Statistics-2018 Update: A Report From the American Heart Association. Circulation.

[12] Nolan JP, Soar J, Smith GB, Gwinnutt C, Parrott F, Power S, Harrison DA, Nixon E, Rowan K (2014). Incidence and outcome of in-hospital cardiac arrest in the United Kingdom National Cardiac Arrest Audit. Resuscitation.

[13] Pearse RM, Moreno RP, Bauer P, Pelosi P, Metnitz P, Spies C, Vallet B, Vincent J, Hoeft A, Rhodes A (2012). Mortality after surgery in Europe: a 7 day cohort study. The Lancet.

[14] Cardona-Morrell M, Prgomet M, Turner RM, Nicholson M, Hillman K (2016). Effectiveness of continuous or intermittent vital signs monitoring in preventing adverse events on general wards: a systematic review and meta-analysis. Int J Clin Pract.

[15] Jones D, Mitchell I, Hillman K, Story D (2013). Defining clinical deterioration. Resuscitation.

[16] Elliott M, Coventry A (2012). Critical care: the eight vital signs of patient monitoring. Br J Nurs.

[17] Appelboom G, Camacho E, Abraham ME, Bruce SS, Dumont EL, Zacharia BE, D'Amico R, Slomian J, Reginster JY, Bruyère O, Connolly ES (2014). Smart wearable body sensors for patient self-assessment and monitoring. Arch Public Health.

[18] Darwish A, Hassanien AE (2011). Wearable and Implantable Wireless Sensor Network Solutions for Healthcare Monitoring. Sensors.

[19] Sahandi R, Noroozi S, Roushan G, Heaslip V, Liu Y (2010). Wireless technology in the evolution of patient monitoring on general hospital wards. J Med Eng Technol.

[20] Subbe CP, Duller B, Bellomo R (2017). Effect of an automated notification system for deteriorating ward patients on clinical outcomes. Crit Care.

[21] Bellomo R, Ackerman M, Bailey M, Beale R, Clancy G, Danesh V, Hvarfner A, Jimenez E, Konrad D, Lecardo M, Pattee KS, Ritchie J, Sherman K, Tangkau P (2012). A controlled trial of electronic automated advisory vital signs monitoring in general hospital wards*. Critical Care Medicine.

[22] Schmidt PE, Meredith P, Prytherch DR, Watson D, Watson V, Killen RM, Greengross P, Mohammed MA, Smith GB (2015). Impact of introducing an electronic physiological surveillance system on hospital mortality. BMJ Qual Saf.

[23] Kollef MH, Heard K, Chen Y, Lu C, Martin N, Bailey T (2017). Mortality and Length of Stay Trends Following Implementation of a Rapid Response System and Real-Time Automated Clinical Deterioration Alerts. Am J Med Qual.

[24] Majumder S, Mondal T, Deen M (2017). Wearable Sensors for Remote Health Monitoring. Sensors.

[25] Khanna AK, Hoppe P, Saugel B (2019). Automated continuous noninvasive ward monitoring: future directions and challenges. Crit Care.

[26] Michard F, Gan T, Kehlet H (2017). Digital innovations and emerging technologies for enhanced recovery programmes. Br J Anaesth.

[27] Fung E, Järvelin Marjo-Riitta, Doshi RN, Shinbane JS, Carlson SK, Grazette LP, Chang PM, Sangha RS, Huikuri HV, Peters NS (2015). Electrocardiographic patch devices and contemporary wireless cardiac monitoring. Front Physiol.

[28] Iqbal MH, Aydin A, Brunckhorst O, Dasgupta P, Ahmed K (2016). A review of wearable technology in medicine. J R Soc Med.

[29] Paton C, Hansen M, Fernandez-Luque L, Lau AYS (2018). Self-Tracking, Social Media and Personal Health Records for Patient Empowered Self-Care. Yearb Med Inform.

[30] Liberati A, Altman DG, Tetzlaff J, Mulrow C, Gøtzsche PC, Ioannidis JPA, Clarke M, Devereaux PJ, Kleijnen J, Moher D (2009). The PRISMA statement for reporting systematic reviews and meta-analyses of studies that evaluate health care interventions: explanation and elaboration. J Clin Epidemiol.

[31] Higgins JPT, Thomas J, Chandler J, Cumpston M, Li T, Page MJ, Welch VA (2019). Cochrane Handbook for Systematic Reviews of Interventions 6th version (updated July 2019). Cochrane.

[32] Joshi M, Ashrafian H, Aufegger L, Khan S, Arora S, Cooke G, Darzi A (2019). Wearable sensors to improve detection of patient deterioration. Expert Rev Med Devices.

[33] Harford M, Catherall J, Gerry S, Young J, Watkinson P (2019). Availability and performance of image-based, non-contact methods of monitoring heart rate, blood pressure, respiratory rate, and oxygen saturation: a systematic review. Physiol Meas.

[34] Craig P, Dieppe P, Macintyre S, Michie S, Nazareth I, Petticrew M, Medical Research Council Guidance (2008). Developing and evaluating complex interventions: the new Medical Research Council guidance. BMJ.

[35] Henrikson N, Skelly A (2013). Economic Studies Part 2: Evaluating the Quality. Evidence-Based Spine-Care Journal.

[36] Hong QN, Pluye P, Fàbregues S, Bartlett G, Boardman F, Cargo M, Dagenais P, Gagnon M-P, Griffiths F, Nicolau B, O’Cathain A, Rousseau M-C, Vedel I (2018). Mixed Methods Appraisal Tool (MMAT), version 2018, Registration of Copyright (#1148552).

[37] Whiting PF (2011). QUADAS-2: A Revised Tool for the Quality Assessment of Diagnostic Accuracy Studies. Ann Intern Med.

[38] Ofman JJ, Sullivan SD, Neumann PJ, Chiou C, Henning JM, Wade SW, Hay JW (2003). Examining the Value and Quality of Health Economic Analyses: Implications of Utilizing the QHES. JMCP.

[39] Prgomet M, Cardona-Morrell M, Nicholson M, Lake R, Long J, Westbrook J, Braithwaite J, Hillman K (2016). Vital signs monitoring on general wards: clinical staff perceptions of current practices and the planned introduction of continuous monitoring technology. Int J Qual Health Care.

[40] Weller RS, Foard KL, Harwood TN (2018). Evaluation of a wireless, portable, wearable multi-parameter vital signs monitor in hospitalized neurological and neurosurgical patients. J Clin Monit Comput.

[41] Izmailova ES, McLean IL, Bhatia G, Hather G, Cantor M, Merberg D, Perakslis ED, Benko C, Wagner JA (2019). Evaluation of Wearable Digital Devices in a Phase I Clinical Trial. Clin Transl Sci.

[42] Breteler MJM, Huizinga E, van Loon K, Leenen LPH, Dohmen DAJ, Kalkman CJ, Blokhuis TJ (2018). Reliability of wireless monitoring using a wearable patch sensor in high-risk surgical patients at a step-down unit in the Netherlands: a clinical validation study. BMJ Open.

[43] Selvaraj N, Nallathambi G, Moghadam R, Aga A (2018). Fully Disposable Wireless Patch Sensor for Continuous Remote Patient Monitoring. Conf Proc IEEE Eng Med Biol Soc.

[44] Liu NT, Holcomb JB, Wade CE, Darrah MI, Salinas J (2014). Evaluation of standard versus nonstandard vital signs monitors in the prehospital and emergency departments. Journal of Trauma and Acute Care Surgery.

[45] Liu NT, Holcomb JB, Wade CE, Darrah MI, Salinas J (2015). Data quality of a wearable vital signs monitor in the pre-hospital and emergency departments for enhancing prediction of needs for life-saving interventions in trauma patients. Journal of Medical Engineering & Technology.

[46] Razjouyan J, Grewal GS, Rishel C, Parthasarathy S, Mohler J, Najafi B (2017). Activity Monitoring and Heart Rate Variability as Indicators of Fall Risk: Proof-of-Concept for Application of Wearable Sensors in the Acute Care Setting. J Gerontol Nurs.

[47] Boatin A, Wylie B, Goldfarb I, Azevedo R, Pittel E, Ng Courtney, Haberer Jessica Elizabeth (2016). Wireless Vital Sign Monitoring in Pregnant Women: A Functionality and Acceptability Study. Telemed J E Health.

[48] Kim J, Roberge R, Powell J, Shafer A, Jon Williams W (2012). Measurement Accuracy of Heart Rate and Respiratory Rate during Graded Exercise and Sustained Exercise in the Heat Using the Zephyr BioHarnessTM. Int J Sports Med.

[49] Van Haren RM, Thorson CM, Valle EJ, Busko AM, Jouria JM, Livingstone AS, Namias N, Schulman CI, Proctor KG (2014). Novel prehospital monitor with injury acuity alarm to identify trauma patients who require lifesaving intervention. Journal of Trauma and Acute Care Surgery.

[50] Meizoso JP, Allen CJ, Ray JJ, Van Haren RM, Teisch LF, Baez XR, Livingstone AS, Namias N, Schulman CI, Proctor KG (2016). Evaluation of Miniature Wireless Vital Signs Monitor in a Trauma Intensive Care Unit. Military Medicine.

[51] Verrillo SC, Cvach M, Hudson KW, Winters BD (2019). Using Continuous Vital Sign Monitoring to Detect Early Deterioration in Adult Postoperative Inpatients. Journal of Nursing Care Quality.

[52] Dur O, Rhoades C, Ng MS, Elsayed R, van Mourik R, Majmudar MD (2018). Design Rationale and Performance Evaluation of the Wavelet Health Wristband: Benchtop Validation of a Wrist-Worn Physiological Signal Recorder. JMIR Mhealth Uhealth.

[53] OrDonnell J, Velardo C, Shah SA, Khorshidi GS, Salvi D, Rahimi K, Tarassenko L (2018). Physical Activity and Sleep Analysis of Heart Failure Patients using Multi-sensor Patches. Conf Proc IEEE Eng Med Biol Soc.

[54] Hubner P, Schober A, Sterz F, Stratil P, Wallmueller C, Testori C, Grassmann D, Lebl N, Ohrenberger I, Herkner H, Weiser C (2015). Surveillance of Patients in the Waiting Area of the Department of Emergency Medicine. Medicine.

[55] Liu Y, Zhu SH, Wang GH, Ye F, Li PZ (2013). Validity and Reliability of Multiparameter Physiological Measurements Recorded by the Equivital Lifemonitor During Activities of Various Intensities. Journal of Occupational and Environmental Hygiene.

[56] Downey C, Brown J, Jayne D, Randell R (2018). Patient attitudes towards remote continuous vital signs monitoring on general surgery wards: An interview study. International Journal of Medical Informatics.

[57] Li T, Divatia S, McKittrick J, Moss J, Hijnen NM, Becker LB (2019). A pilot study of respiratory rate derived from a wearable biosensor compared with capnography in emergency department patients. OAEM.

[58] Paul JE, Chong MA, Buckley N, Harsha P, Shanthanna H, Tidy A, Buckley D, Clarke A, Young C, Wong T, Vanniyasingam T, Thabane L (2019). Vital sign monitoring with continuous pulse oximetry and wireless clinical notification after surgery (the VIGILANCE pilot study)-a randomized controlled pilot trial. Pilot Feasibility Stud.

[59] Weenk M, van Goor H, Frietman B, Engelen LJ, van Laarhoven CJ, Smit J, Bredie SJ, van de Belt TH (2017). Continuous Monitoring of Vital Signs Using Wearable Devices on the General Ward: Pilot Study. JMIR Mhealth Uhealth.

[60] Watkins T, Whisman L, Booker P (2016). Nursing assessment of continuous vital sign surveillance to improve patient safety on the medical/surgical unit. J Clin Nurs.

[61] Hernandez-Silveira M, Wieczorkowski-Rettinger K, Ang S, Burdett A (2015). Preliminary assessment of the SensiumVitals®: A low-cost wireless solution for patient surveillance in the general wards. Conf Proc IEEE Eng Med Biol Soc.

[62] Downey C, Randell R, Brown J, Jayne DG (2018). Continuous Versus Intermittent Vital Signs Monitoring Using a Wearable, Wireless Patch in Patients Admitted to Surgical Wards: Pilot Cluster Randomized Controlled Trial. J Med Internet Res.

[63] Hernandez-Silveira M, Ahmed K, Ang S, Zandari F, Mehta T, Weir R, Burdett A, Toumazou C, Brett SJ (2015). Assessment of the feasibility of an ultra-low power, wireless digital patch for the continuous ambulatory monitoring of vital signs. BMJ Open.

[64] Downey C, Ng S, Jayne D, Wong D (2019). Reliability of a wearable wireless patch for continuous remote monitoring of vital signs in patients recovering from major surgery: a clinical validation study from the TRaCINg trial. BMJ Open.

[65] Chan AM, Selvaraj N, Ferdosi N, Narasimhan R (2013). Wireless patch sensor for remote monitoring of heart rate, respiration, activity, and falls. Annual International Conference of the IEEE Engineering in Medicine and Biology Society (EMBC).

[66] Sotera W Sotera Wireless.

[67] Sensium Healthcare (2019). Early detection of patient deterioration.

[68] VitalConnect (2017). The VitalConnect Solution.

[69] Liu NT, Holcomb JB, Wade CE, Darrah MI, Salinas J (2014). Evaluation of standard versus nonstandard vital signs monitors in the prehospital and emergency departments. Journal of Trauma and Acute Care Surgery.

[70] Athena GTX (2019). Published.

[71] Athena GTX Product catalog.

[72] Medtronic Medtronic.

[73] Philips (2019). Published.

[74] Wavelet H (2019). Published.

[75] Proteus Digital Health, Inc (2019). Proteus Digital Health Feedback Device.

[76] Philips Healthcare (2013). Published.

[77] Hildago Ltd. (2012). Published.

[78] Mochizuki K, Shintani R, Mori K, Sato T, Sakaguchi O, Takeshige K, Nitta K, Imamura H (2017). Importance of respiratory rate for the prediction of clinical deterioration after emergency department discharge: a single-center, case-control study. Acute Med Surg.

[79] Flenady T, Dwyer T, Applegarth J (2017). Accurate respiratory rates count: So should you!. Australas Emerg Nurs J.

[80] Parkes R (2011). Rate of respiration: the forgotten vital sign. Emerg Nurse.

[81] Yonge JD, Bohan PK, Watson JJ, Connelly CR, Eastes L, Schreiber MA (2018). The Respiratory Rate: A Neglected Triage Tool for Pre-hospital Identification of Trauma Patients. World J Surg.

[82] Mendt S, Maggioni MA, Nordine M, Steinach M, Opatz O, Belavý Daniel, Felsenberg D, Koch J, Shang P, Gunga H, Stahn A (2017). Circadian rhythms in bed rest: Monitoring core body temperature via heat-flux approach is superior to skin surface temperature. Chronobiol Int.

[83] Henriksson E, Lamia KA (2015). Adipose Clocks: Burning the Midnight Oil. J Biol Rhythms.

[84] Albrecht U (2012). Timing to perfection: the biology of central and peripheral circadian clocks. Neuron.

[85] Slight SP, Franz C, Olugbile M, Brown HV, Bates DW, Zimlichman E (2014). The Return on Investment of Implementing a Continuous Monitoring System in General Medical-Surgical Units*. Critical Care Medicine.

[86] Downey C, Chapman S, Randell R, Brown J, Jayne D (2018). The impact of continuous versus intermittent vital signs monitoring in hospitals: A systematic review and narrative synthesis. Int J Nurs Stud.

[87] Churpek MM, Adhikari R, Edelson DP (2016). The value of vital sign trends for detecting clinical deterioration on the wards. Resuscitation.

[88] Escobar GJ, LaGuardia JC, Turk BJ, Ragins A, Kipnis P, Draper D (2012). Early detection of impending physiologic deterioration among patients who are not in intensive care: development of predictive models using data from an automated electronic medical record. J Hosp Med.

[89] Alam N, Hobbelink E, van Tienhoven A, van de Ven P, Jansma E, Nanayakkara P (2014). The impact of the use of the Early Warning Score (EWS) on patient outcomes: a systematic review. Resuscitation.

[90] Sendelbach S, Funk M (2013). Alarm Fatigue. AACN Advanced Critical Care.

[91] Kowalski R, Capan M, Lodato P, Mosby D, Thomas T, Arnold R, Miller K (2017). Optimizing usability and signal capture: a proactive risk assessment for the implementation of a wireless vital sign monitoring system. J Med Eng Technol.

[92] Frost&Sullivan Topline Finding Top-Line Opportunities in Bottom- line HC Market.

