# Supplementary material for: Current Evidence for Continuous Vital Signs Monitoring by Wearable Wireless Devices in Hospitalized Adults: Systematic Review
Source: J Med Internet Res. 2020 Jun 17;22(6):e18636. doi: 10.2196/18636 (PMC7351263; doi:10.2196/18636)
Supplement: Multimedia Appendix 1 [file jmir_v22i6e18636_app1.docx]

**Multimedia Appendix 1: search string of database PubMed/Medline**

#1 vital OR vital sign* OR vital function* OR vital parameter* OR clinical deterioration OR deterioration OR Vital Signs [MeSH]

#2 Remote continuous monitoring OR Wireless continuous monitoring OR wireless device OR patch OR appliance OR wearable OR portable OR smart OR sensor OR Physiologic Monitoring [MeSH]

#3 clinical outcome OR mortality OR death OR length of stay OR LoS OR readmission OR intensive care unit admission OR ICU admission OR rapid response team OR RRT OR intervention* OR sepsis OR operation OR valid* OR reliab* OR feasibility* OR acceptability OR demand OR implementation OR practicality OR adaptation OR integration OR expansion OR limited-efficacy testing OR cost* OR cost-effectiveness OR cost-efficient
